# Leptin receptor gene polymorphisms *c.668A>G* and *c.1968G>C* in Sudanese women with preeclampsia: a case-control study

**DOI:** 10.1186/s12881-020-01104-z

**Published:** 2020-08-17

**Authors:** Amira Saad, Ishag Adam, Salah Eldin G. Elzaki, Hiba A. Awooda, Hamdan Z. Hamdan

**Affiliations:** 1 Department of Biochemistry and Molecular Biology, Faculty of Medicine, Al-Neelain University, PO BOX 12702, Khartoum, Sudan; 2 Department of Obstetrics and Gynecology, Unaizah College of Medicine and Medical Sciences, Qassim University, Unaizah, Kingdom of Saudi Arabia; 3 Department of Epidemiology, Tropical Medicine Research Institute, Khartoum, Sudan; 4 Department of Basic Medical Sciences, Unaizah College of Medicine and Medical Sciences, Qassim University, Unaizah, Kingdom of Saudi Arabia

**Keywords:** Preeclampsia, Leptin receptor gene, Polymorphism, Sudan, Haplotype

## Abstract

**Background:**

Leptin receptor gene (*LEPR*) variants may affect the leptin levels and act as a risk factor for preeclampsia. Two *LEPR* gene missense variants rs1137101 *(c.668A>G)* and rs1805094 *(c.1968G>C)* were investigated in Sudanese women with preeclampsia.

**Methods:**

A matched case-control study (122 women in each arm) was conducted in Saad Abualila Maternity Hospital in Khartoum, Sudan from May to December 2018. The cases were women with preeclampsia and the controls were healthy pregnant women. Genotyping for *LEPR* gene variants *c.668A>G* and *c.1968G>C* was performed using polymerase chain reaction-restriction fragment length polymorphism. Logistic regression models (adjusted for age, parity, body mass index and hemoglobin level) were conducted.

**Results:**

Genotype frequency of *LEPR* gene variants *c.668A>G* and *c.1968G>C* was in accordance with Hardy–Weinberg equilibrium (*P* > 0.05) in the controls. Allele G in *LEPRc.668A>G* variant was significantly more frequent in the cases compared with the controls [43.4% vs. 10.2%; OR = 6.44; 95%CI (3.98–10.40); *P* < 0.001]. In variant *LEPRc.668A>G*, genotype AG was the prevalent genotype in the cases compared with the controls, and it was significantly associated with preeclampsia risk [37.7% vs. 15.5%; AOR = 3.48; 95%CI (1.15–10.54); *P =* 0.027]. Likewise, the GG genotype was the second most common genotype in the cases compared with the controls, and was associated with preeclampsia risk [24.6% vs. 2.5%; AOR = 14.19; 95%CI (1.77–113.76); *P* = 0.012]. None of the *LEPRc.1968G>C* variant genotypes were associated with preeclampsia. The CC genotype was not detected in neither the cases nor the controls. The haplotype A-G 70.1% was the prevalent haplotype in this population, and it significantly protected against preeclampsia [OR = 0.14; 95%CI (0.09–0.23); *P* < 0.001]. However, the haplotype G-G 26.8% was significantly associated with preeclampsia risk [OR = 6.70; 95%CI (4.16–11.05); *P* < 0.001]. Both variants *c.668A>G* and *c.1968G>C* were in strong linkage disequilibrium (D′ = 1, *r*^2^ = 0.012).

**Conclusions:**

Our data indicate that the rs1137101 (*c.668A>G)* variant and G-G haplotype may independently associate with the development of preeclampsia.

## Background

Preeclampsia, is a common pregnancy related disorder characterized by new-onset of hypertension and proteinuria after the 20th week of gestation [1]. It affects approximately 2–8% of pregnancies worldwide [2, 3]. Preeclampsia is a major cause for maternal and foetal morbidity and mortality. It can lead to dysfunction of multiple systems and organs including the liver, kidneys and brain [3]. Although its aetiology is not well established, a multifactorial model with major genetic involvement has been previously mooted [4].

Many reports suggest that preeclampsia is associated with high serum and placental leptin levels [5, 6]. Leptin is an adipocyte secreting hormone that control appetite and weight gain [7]. In pregnancy, the placenta and syncytio-trophoblasts are also able to synthesize and secrete leptin [7, 8]. In addition to its action in controlling body energy, in the placenta, leptin controls angiogenesis, smooth muscle proliferation and modulates the release of many inflammatory mediators and cytokines [9, 10]. These provide the rationale for endothelial dysfunction that is implicated in the pathogenesis of preeclampsia [11]. However, a previous study indicate that leptin has no role in the pathogenesis of preeclampsia [12] and the increment in placental leptin expression may be attributed to the adaptive mechanism to hypoxia observed in preeclampsia [13].

Leptin exerts its functions through its receptor (LEPR) [14]. LEPR belongs to cytokine receptor superfamily and is expressed in many tissues, including the placenta [15]. It is a product of the leptin receptor gene (*LEPR,* OMIM: 601007), which is mapped to chromosome 1 in the cytogenetic band 1p31.3 [16]. Single nucleotide polymorphisms (SNPs) sequenced to the *LEPR* gene, such as rs1137101 (*LEPR c.668A>G*) and rs1805094 (*LEPR c.1968G>C*), may have structural and functional effects in LEPR [17]. Both these SNPs are associated with the development of preeclampsia [18, 19]. However, many other studies have reported no association [20, 21]. Chromosome 1 contains many loci associated with preeclampsia, namely, *MTHFR* gene 1p36.3, *FV* gene 1q23 and *LEPR* gene 1p31.3 [18, 22, 23].

A dearth of studies have investigated the association between *LEPR* variants rs1137101 and rs1805094 and preeclampsia worldwide [18–21]. However, all these studies reported on either Caucasian or Asian groups [19, 21]. To the best of our knowledge, no report has investigated the frequency and association between rs1137101 or rs1805094 and preeclampsia among sub Saharan African patients, including those in Sudan. Therefore, this study investigated the association of rs1137101 and rs1805094 in the development of preeclampsia among Sudanese patients and adds to our previous reports on the genetics of preeclampsia in Sudan [22–24].

## Methods

This was a matched (for age and parity with ±1 for the age and parity) case-control study (122 participants in each arm) conducted at Saad Abualila Maternity Hospital, Khartoum, Sudan from May to December 2018. All participants were Sudanese women descended from northern part of Sudan who spoke Arabic language [25]. The cases were women presented with preeclampsia in the second half of pregnancy. Preeclampsia was diagnosed according to American College of Obstetricians and Gynaecologists criteria [1]. Cases with other co-morbidities beside preeclampsia like diabetes mellitus, gestational diabetes, thyroid diseases, and haemolytic disease were excluded. Briefly, pregnant women with blood pressure ≥ 140/90 mmHg on two occasions, at least 6 h apart, and proteinuria of ≥300 mg/24 h were considered to have preeclampsia. Preeclampsia was further described as either mild or severe. Severe preeclampsia was defined as a blood pressure ≥ 160/110 mmHg on two occasions, proteinuria of ≥5 g/24 h, and HELLP syndrome (hypertension, proteinuria and presence of haemolytic anaemia, elevated liver enzymes, and low platelet count) [1]. Early onset and late onset preeclampsia were terms if preeclampsia confirmed before and after 34 weeks of gestation, respectively [26]. Healthy pregnant women without any underlying diseases such as cardiovascular diseases including hypertension, renal diseases, diabetes or proteinuria were considered as controls. Both the cases and the controls recruited from labour ward.

The participants’ basic clinical characteristics and obstetrics information (age, number of pregnancies, and gestational age) were collected by one of the investigators using a questionnaire. Body mass index (BMI) in early pregnancy (< 14 weeks of gestational) was computed as weight (Kg)/Height(m^2^). Based on World Health Organization (WHO) classification, BMI was classified into normal weight (18.5–24.9 kg/m^2^), overweight (25.0–29.9 kg/m^2^) and obese (≥30.0 kg/m^2^) [27]. After that, for each participant, 3 ml of whole blood was collected in vacutainers containing anticoagulant (EDTA) for DNA extraction and haemoglobin estimation. Genomic DNA was isolated using QIAamp DNA Blood kits (Qiagen) following the manufacturer’s instructions. Haemoglobin was measured immediately by using portable HemoCue® hemoglobinometer (HemoCue® AB, Ängelhom, Sweden).

### SNP genotyping

The *LEPR c.668A>G* (rs1137101, also known as *Q223R*) and *LEPR c.1968G>C* (rs1805094, also known as *K656N*) polymorphisms were determined by examining the length of the polymerase chain reaction (PCR) products generated by DNA amplification of the *LEPR* gene (NG_015831.2) target sequences in exons 6 and 14 for the *LEPR c.668A>G* (rs1137101) and *LEPR c.1968G>C* **(**rs1805094) polymorphisms, respectively. The result of *LEPR* genotypes were confirmed further by sequencing the PCR products using the same primer set for both variants (sequencing was done by Macrogen Inc., South Korea). Details of the primers set and restriction enzymes used for PCR and the polymerase chain reaction-restriction fragment length polymorphism (PCR-RFLP) digestion results for the *LEPR* polymorphisms are shown in Table S1. All PCR reactions were done in 25-μl volumes that contained 5 μl purified DNA, 1 μl each forward and reverse primer, 5 μl reaction mixture and 13 μl of ultrapure water. For detection of the *LEPR c.668A>G* (rs1137101), the PCR settings were as follow: initial denaturation at 95 °C for 2 min, 35 cycles of denaturation at 95 °C for 30 s, annealing at 54 °C for 45 s, extension at 72 °C for 45 s, and final extension at 72 °C for 10 min, as previously described [28]. For *LEPR c.1968G>C* (rs1805094), the settings were as follow: initial denaturation at 94 °C for 5 min, 35 cycles of denaturation at 94 °C for 30 s, annealing at 49 °C for 45 s, extension at 72 °C for 1 min, and final extension at 72 °C for 5 min, as described before [29]. Afterwards, the PCR product was digested by restriction enzymes *MspI* and *BstU1* for *LEPR c.668A>G* (rs1137101) and *LEPR c.1968G>C* (rs1805094), respectively (New England BioLabs® Inc.). The mixture (PCR product and restriction enzyme) was incubated overnight at 37 °C in a 21-μl reaction volume. Then, digested fragments were parted using 3% agarose gel electrophoresis and marked with ethidium bromide, look at Figure S1. Details of the PCR-RFLP genotyping are shown in Table S1.

### Sample size calculation

The sample size of 122 participants was calculated based on a 1:1 ratio of the cases to the controls and the expected difference in the rate of rs1137101 polymorphism between the two groups. Based on a previous report, we assumed that rs1137101 would be found in 35% of healthy pregnant controls, and in 54% of pregnant women with preeclampsia [30]. This would provide the study an 80% power to detect differences of 0.05 at the α-level.

### Statistics

The collected data were entered into computer using SPSS for Windows for data analysis. The clinical data were compared between the cases and controls using Student’s *t-* tests and Pearson’s chi-square (χ^2^) tests for continuous and categorical data, respectively. The observed and expected genotype distributions and their agreement with Hardy–Weinberg equilibrium (HWE) were evaluated by using the chi-square (χ^2^) goodness-of-fit test. Differences in allele frequencies between the cases and controls were compared by Pearson’s chi-square (χ^2^) statistical test. The risk associated with a specific genotype was initially estimated by univariate analysis (Chi-square or Fisher’s exact test) and odds ratios, and is expressed with a 95% confidence interval.

Multiple logistic regression (backward conditional) models were then applied with preeclampsia as dependent variable and maternal age, parity, BMI, hemoglobin and genotypes of *LEPR c.668 A > G (Q223R)* and *LEPR c.1968G>C* (*K656N*) as independent variables. The adjusted odds ratio, 95% confidence interval, and the *p*-value were reported. Haplotype frequencies and tests for LD between *LEPR c.668A>G* and *LEPR c.1968G>C* were performed using Haploview v4.2 (www.broad.mit.edu/mpg/haploview/). Allele frequencies in our study population were compared with other African populations, a Luhya population in Kenya and a Yourba population in Nigeria, the results of which were retrieved from 1000 Genomes Project data [31]. Global allele frequencies for rs1137101 and rs1805094 were retrieved from dbSNP-refSNP [32, 33]. A *p*-value < 0.05 considered statistically significant at two-sides. The dataset generated and analysed for the current study is included as supplementary file (Supplemental dataset).

## Results

There were no significant differences in the age, parity, gestational age, BMI or haemoglobin levels between the cases and the controls are shown in Table 1.

**Table 1:** The mean (SD) or n (%) of the basic characteristics between women with preeclampsia and the controls

Variables	Preeclampsia ***N*** = 122	Controls ***N*** = 122	***P-***value
**Age, year**	27.4 (2.2)	27.1 (2.1)	0.609
**Parity**	2.7 (1.4)	2.8 (1.5)	0.449
**Gestational age, weeks**	37.4 (1.7)	37.6 (1.9)	0.226
**Systolic blood pressure, mm/Hg**	148.7 (11.0)	134.4 (21.7)	< 0.001
**Diastolic blood pressure, mm/Hg**	98.1 (6.7)	89.6 (7.6)	< 0.001
**Hemoglobin, g/dl**	10.8 (1.8)	10.9 (1.9)	0.552
**Body mass index, Kg/m**^**2**^	24.8 (1.7)	24.9 (1.8)	0.530
**Body mass index categories**			
***Under-weight***	5 (4.1)	3 (2.5)	
***Normal***	65 (53.3)	62 (50.8)	
***Over-weight***	41 (33.6)	37 (30.3)	0.348
***Obese***	11 (9)	20 (16.4)	

Forty–two (34.4%) and 80 (65.5%) women had severe and mild preeclampsia, respectively. Sixteen (13.11%) and 106 (86.88%) women presented with early preeclampsia and late preeclampsia, respectively.

Distribution of the genotypes and alleles of *LEPR c.668A>G* (rs1137101) (goodness of fit χ^2^ = 2.8641, df = 1, *P* > 0.05 and *LEPR c.1968G>C* (rs1805094) (goodness of fit χ^2^ = 0.033, df = 1, *P* > 0.05) in the controls was in accordance with the Hardy–Weinberg equilibrium.

The *LEPR c.668A>G* AG [(37.7% vs. 15.57%), AOR = 3.48, 95%CI = 1.15–10.54; *P* = 0.027] and GG [(24.6% vs. 2.5%), AOR = 14.19, 95%CI = 1.77–113.76; *P* = 0.012] genotype was associated with the risk of preeclampsia (AA genotype was a reference), Table 2. Moreover, the proportion of the G allele was significantly higher in women with preeclampsia compared with the proportion in healthy pregnant women [(43.4% vs. 10.2%), OR = 6.44, 95%CI = 3.98–10.40; *P* < 0.001].

**Table 2:** Comparing the genotypes and alleles of *LEPR c.668 A > G (Q223R)* and *LEPR c.1968G>C* (*K656N)* between women with preeclampsia and the controls

Genotypes	Preeclampsia (122)		Controls (122)		Crude OR (95% CI)	***P***-value	^**a**^OR (95% CI)	***P***-value
	N	%	N	%				
***LEPR c.668 A > G***								
**AA**	46	37.7	100	81.96	Reference		Reference	
**AG**	46	37.7	19	15.57	4.41 (1.32–16.66)	0.016	3. 48 (1.15–10.54)	0.027
**GG**	30	24.6	3	2.5	16.65 (1.93–143.50)	< 0.001	14.19 (1.77–113.76)	0.012
***LEPR c.1968G>C***								
**GG**	111	91.0	118	96.7	Reference		Reference	
**GC**	11	9.0	4	3.3	1.32 (0.81–2.13)	0.258	3.08 (0.35–26.58)	0.350
**CC**	0	0	0	0	–	–	–	–

Note: *LEPR* (NG_015831.2). ^a^adjusted odd ratio for maternal age, parity, BMI and hemoglobin

The distribution of the *LEPR c.668A>G* AG genotype was not significantly different between women with severe and mild preeclampsia [16/42 (38.09%) vs. 30/80 (37.5%); *P* = 0.984] or women with early and late preeclampsia [7/16 (43.75%) vs. 39/106 (36.79%); *P* = 0.796]. Moreover, the distribution of *LEPR c.668A>G* GG genotype was not significantly different between women with severe and mild preeclampsia [10/42 (23.80%) vs. 20/80 (25.0%); *P* = 0.939] or women with early and late preeclampsia [6/16 (37.50%) vs. 24/106 (22.6%); *P* = 0. 329].

Although the *LEPR c.1968G>C* GC [(9.01% vs. 3.3%), AOR = 3.08, 95%CI = 0.35–26.58; *P* = 0.350] genotype was not associated with the risk of preeclampsia, the *LEPR c.1968G>C* CC genotype was not detected in any of the studied women in either the case or control groups (GG genotype was a reference). The proportion of the C allele was higher in women with preeclampsia compared with the proportion in healthy pregnant women, however it did not reach statistical significance [(4.5% vs. 1.6%), OR = 2.83, 95%CI = 0.91–11.1; *P* = 0.072].

The distribution of *LEPR c.1968G>C* GC genotype was not significantly different between women with severe and mild preeclampsia [4/42 (9.52%) vs. 9/80 (11.25%); *P* = 0.987] or women with early and late preeclampsia [2/16 (12.5%) vs. 11/106 (10.37%); *P* = 0.858].

Table 3 shows a comparison of allele frequencies of both variants *c.668A>G* and *c.1968G>C* in our studied populations and other studies from Hungary, Germany, Sri -Lanka, and China [18–21, 34]. The frequency of allele G in the variant *c.668A>G* in our study group (26.9) was slightly lower than in the Sri-Lankan population (35.8) and much lower than in the global allele frequency (44.9), Hungarian population (42.5) and German population (57.3). In variant *c.1968G>C* the frequency of C allele in study group (3.1) was lower than in the Hungarian population (17.8), the global allele frequency (19.1) and Chinese population (23.9).

**Table 3:** Comparison of allele frequency of the *LEPR c.668 A > G* and *LEPR c.1968G>C* polymorphisms in the Sudanese sample with world population

Population	***LEPR c.668 A > G*** rs1137101		***LEPR c.1968G>C*** rs1805094	
	A	G	G	C
**Sudanese.** (the current study)	73.1	26.9	96.9	3.1
**Hungarian.** Rigó J. *et. al.,* 2006 [18].	57.5	42.5	–	–
**Hungarian.** Várkonyi *et. al.,* 2010 [21].	43.0	57.0	82.2	17.8
**Sri-Lankan.** Tennekoon *et. al.,* 2012 [34].	64.1	35.8	–	–
**Chinese.** Wang *et. al.,* 2013 [19].	–	–	76.0	23.9
**German.** Wiedemann *et. al.,* 2010 [20].	42.6	57.3	–	–
**Global allele frequency.**(dbSNP-refSNP) [32, 33]	55.0	44.9	80.8	19.1

Haplotype analysis for the two variants *LEPR c.668A>G* and *LEPR c.1968G>C* identified three haplotypes: A-G at 70.1%, which was the most frequent, followed by G-G at 26.8% and A-C at 3.1%. The G-C haplotype was not detected, Table 4. A strong LD was observed between *c.668A>G* and *c.1968G>C* (D′ = 1, *r*^2^ = 0.012), Figure S2. The A-G haplotype frequency versus other pooled haplotypes (G-G and A-C) was significantly lower in the cases than in the controls [OR = 0.14, 95%CI = 0.09–0.23; *P* < 0.001], while, the G-G haplotype frequency versus other pooled haplotypes (A-G and A-C) was significantly higher in cases than in the controls [OR = 6.70, 95%CI = 4.16–11.05; *P* < 0.001].

**Table 4:** Detailed haplotypes frequencies of the *LEPR c.668 A > G* and *LEPR c.1968G>C* variants in the pre-eclamptic patients and controls

Haplotype	***Total***	***Cases*** *N* = 244	***Controls*** *N* = 244
**A-G**	342 (70.1)	127 (52)	215 (88.1)
**G-G**	131 (26.8)	106 (43.4)	25 (10.2)
**A-C**	15 (3.1)	11 (4.5)	4 (1.6)

Data are expressed as n(%), where n denotes the number of chromosomes examined

## Discussion

In this study we investigated the relationship between the two variants *LEPR c.668 A > G* and *LEPR c.1968G>C* and the development of preeclampsia among Sudanese women. The major finding in this study was the significant association between *LEPR* variant c*.668A>G* and preeclampsia. Our finding was in accordance with the findings of Rigo et al. in a study of pregnant Hungarian women [18]. However, several other studies showed no association between c*.668A>G* and preeclampsia [20, 21, 34, 35]. In this study, the frequency of the minor G allele, whether it was the AG or GG genotype, was significantly associated with the development of preeclampsia. Moreover, the minor allele frequency (MAF) in this study was G = 0.269. This was close to the 0.358 value that was reported in a study of a Sri Lankan population [34]. Although a significant association between *LEPR* c*.668A>G* and preeclampsia was reported only by Rigo *et. al.* in a study of Hungarian women, the G allele frequency that Rigo’s team reported was 0.425 [18]. Another study in a Hungarian population showed that the MAF was 0.57, which is nearly identical to the 0.573 that was reported by Wiedemann *et. al.,* in a German study [20, 21]. No investigation of an African population regarding the *LEPR* c*.668A>G* association with preeclampsia has been previously conducted. According to the 1000 Genomes Project, the MAF among African populations is the A allele, and the closest MAF what we found is G = 0.48, which was observed in a Luhya population in Kenya [31]. This variation in the MAF observed in an African population is not entirely unexpected because African populations are characterized as having high genetic diversity within and between other populations [36], although there is more obvious heterogeneity within the Sudanese population [37]. It is worth noting that the MAF in our study was more than 0.1, which explains the implication of this variant in the association with preeclampsia in our population. Furthermore, a genome wide association study reported that the *LEPR* c*.668A>G* variant was significantly associated with a decrease in leptin receptor levels among women of European descent [38]. It has also been reported that the *LEPR* c*.668A>G* variant is associated with increased serum leptin levels among healthy female adolescents [39]. A very recent systematic review reported a significant association of high serum leptin levels with preeclampsia, regardless of the onset of preeclampsia or its severity [40]. In addition, a gene microarray study demonstrate that a high expression of the leptin gene is present in pregnant women with preeclampsia [41]. At the molecular level, *LEPR* c*.668A>G* has a substitution of the A nucleotide from the G nucleotide at + 668 in exon 6. This substitution changed the wild amino acid glutamine to arginine at position 223 in the leptin receptor protein [31]. Biophysically, glutamine is a neutral amino acid, but arginine is a positively charged amino acid. Moreover, arginine is far bigger than the wild amino acid glutamine [42]. These differences between the wild and mutant amino acids may affect the stability of the 3D structure of the leptin receptor and in turn decrease its levels in the circulating blood. The Bioinformatic tool *I-mutant-2* predicted the structure of the leptin receptor as an unstable protein when bound with the mutant amino acid arginine [43]. In light of these premises, our result suggests an independent association of *LEPR* c*.668A>G* variant with the development of preeclampsia in Sudanese women.

In this study, we did not detect a significant difference in the genotypes or allele frequencies of the *LEPR c.1968G>C* variant in women with preeclampsia compared with controls. However, the frequency of allele C, which is a minor allele, was higher among the cases compared with the controls. This finding is in agreement with a study from Hungary [21]. Furthermore, Wang *et. al.,* reported a significant association of *LEPR c.C1968 homozygous + LEPR c.G1968 heterozygous* with preeclampsia in a Chinese population [19]. The *LEPR c.1968G>C* variant led to a change of G to C at nucleotide c.1968 in exon 14. This substitution lead to change of the wild amino acid lysine to asparagine at codon 656 [31]. Biophysically, the wild amino acid is wide and positively charged, but the mutant is small and has a neutral charge [40]. Such differences in the physical properties between the wild and mutant amino acids may decrease the stability of the receptor and affect its function [43]. Wang *et. al.* found significantly high leptin and insulin levels in patients who have the *c.C1968 homozygous + c.G1968 heterozygous* variants. This confirmed that *LEPR c.G1968* is a functional variant with a possible damaging effect [19]. Perhaps the *LEPR c.1968G>C* variant is the direct cause of the insulin resistance observed in the study by Wang *et. al.,* and possibly mediates the development of preeclampsia during pregnancy [19]. Insulin resistance is a major risk for developing preeclampsia, and this is manifested through many ways. First, hyperinsulinemia directly increases sodium reabsorption in the kidneys and the sympathetic nervous system tone. Second, hyperglycaemia exaggerates the endothelial dysfunction found in the placenta. Third, high leptin levels may promote preeclampsia through altering insulin levels, signalling transduction, reaction with other proteins and metabolism [19, 44, 45].

In this study, the two variants c*.668A>G* and *c.1968G>C* were found to be in strong LD (D′ = 1), which is very close to the D′ = 0.934 that was reported by Várkonyi *et. al.* in Hungarian patients [21], yet they are non-tagging to each other’s *r*^2^ = 0.012. This may be explained by the very low frequency of the C allele (0.031) and the absent GC haplotype observed in this study. Our result is in line with that observed in some African populations, namely, a Luhya population in Kenya (D′ = 0.847; *r*^2^ = 0.172) and a Yourba population in Nigeria (D′ = 0.690; *r*^2^ = 0.081) [31]. The A-G haplotype was the most frequent haplotype in our population, and it showed a protective effect against the development of preeclampsia. However, the G-G haplotype was significantly associated with preeclampsia risk. This further confirmed the involvement of the G allele in the *LEPR c.668A>G* variant as an allele associated with the risk of developing preeclampsia.

This study is the first study conducted in A Sub-Saharan African population; however, it contains many limitations. First, we didn’t investigate the LEPR gene expression. Second, leptin levels were not measured. Third, although sample size is calculated with 80% power, the C allele in the c*.1968G>C* frequency is very low. Therefore, further study is needed with more sample size to trace the missing haplotype and measure the expression of *LEPR* gene and leptin levels.

## Conclusions

To sum up, the current study showed that G allele in *LEPR c.668A>G* and haplotype G-G are associated with development of preeclampsia. The haplotype A-G is the most prevalent haplotype in our population and showed a protective effect against preeclampsia. Strong LD has been observed between the two variants *c.668A>G* and *c.1968G>C*.

## Supplementary information

**Additional file 1 Table S1.** Oligonucleotides, restriction enzymes and RFLP used for detection of *LEPR* gene polymorphisms. **Figure S1.** Lanes 3, 4,8 and 9: heterozygous; lanes 2 and 5: homozygous for wild type allele (421 bp); lanes 6 and 10: homozygous for mutant type allele (294 bp and 27 bp). Lane M contains the 100 bp DNA molecular weight marker; lanes 1 and 7 negative control. **Figure S2.** Haplotype analysis of the two loci LEPR c.668A>G and c.1968G>C studied. Linkage disequilibrium (LD) of the *LEPR* gene observed between loci *c.668A>G* and *c.1968G>C* (D’ = 1; *r*^2^ = 0.012).

**Additional file 2.** Supplemental Dataset

## Data Availability

The dataset generated and/or analysed during the current study are available in the ResearchGate repository: https://www.researchgate.net/publication/343367085_Amira_Saed_Ishag_Adam_Salah_Eldin_G_Elzaki_Hiba_A_Awooda_Hamdan_Zaki_Hamdan_Leptin_receptor_gene_polymorphisms_c668AG_and_c1968GC_in_Sudanese_women_with_preeclampsia_a_case-control_study_Data_set. DOI:10.13140/RG.2.2.26798.95046 The NG_015831.2 dataset generated and/or analyzed during the current study are available in the NIH genetic sequence database (NCBI GeneBank): https://www.ncbi.nlm.nih.gov/genbank/

## Abbreviations

BMI: Body Mass Index
EDTA: Ethylene Diamine Tetra Acetic Acid
HWE: Hardy–Weinberg Equilibrium
HELLP syndrome: Hemolysis, Elevated Liver Enzymes, Low Platelet Count
LD: Linkage disequilibrium
LEPR: Leptin receptor
*LEPR*: Leptin receptor gene
PCR: Polymerase Chain Reaction
PCR–RFLP: Polymerase Chain Reaction–Restriction Fragment Length Polymorphism
TBE: Tris Base/Borate/EDTA
UV: Ultra Violet
WHO: World Health Organization
