# Supplementary material for: Leptin receptor gene polymorphisms c.668A>G and c.1968G>C in Sudanese women with preeclampsia: a case-control study
Source: BMC Med Genet. 2020 Aug 17;21:162. doi: 10.1186/s12881-020-01104-z (PMC7433111; doi:10.1186/s12881-020-01104-z)
Supplement: Supplementary file 1 — Additional file 1 Table S1. Oligonucleotides, restriction enzymes and RFLP used for detection of LEPR gene polymorphisms. Figure S1. Lanes 3, 4,8 and 9: heterozygous; lanes 2 and 5: homozygous for wild type allele (421 bp); lanes 6 and 10: homozygous for mutant type allele (294 bp and 27 bp). Lane M contains the 100 bp DNA molecular weight marker; lanes 1 and 7 negative control. Figure S2. Haplotype analysis of the two loci LEPR c.668A>G and c.1968G>C studied. Linkage disequilibrium (LD) of the LEPR gene observed between loci c.668A>G and c.1968G>C (D’ = 1; r2 = 0.012). [file 12881_2020_1104_MOESM1_ESM.docx]

**Table S1**: **Oligonucleotides, restriction enzymes and RFLP used for detection of *LEPR* gene polymorphisms**

| ***LEPR* gene Polymorphism** | **rs identifier and genomic coordinate** | **Primers pair** | **Restriction enzyme used** | **Fragments length**  **(bp)** |
| --- | --- | --- | --- | --- |
| ***LEPR c.668A>G***  ***Q223R*** | **rs1137101**  chromosome 1:65592830 | Fwd. 5’-ACCCTTTAAGCTGGGTGTCCCAAATAG-3’  Rev. 5’-AGCTAGCAAATATTTTTGTAAGCAATT-3’ | *MspI* | GG: 294+127  AG: 21+294+127  AA: 421 |
| ***LEPRc.1968G>C***  ***K656N*** | [**rs1805094**](https://www.ncbi.nlm.nih.gov/snp/rs1805094)  chromosome 1:65610269 | Fwd. 5’-ACTAGATGGACTGGGATATTGGAGTAAT-3’  Rev. 5’CTTCCAAAGTAAAGTGACATTTTTCGC-3’ | *BstU1* | GG: 251  GC: 251+231  CC:231 |

Note, Fwd: forward; Rev: reverse; *LEPR* (NG_015831.2).


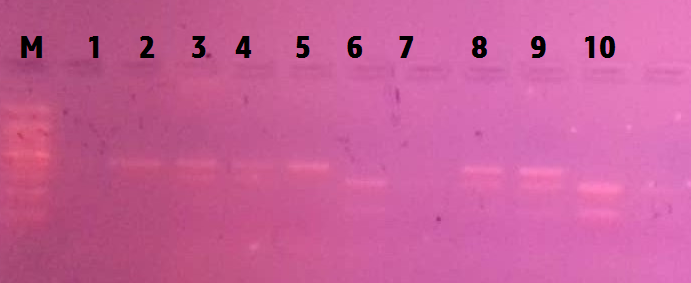


421bp

294bp

127bp

**Figure S1. *LEPR c.668A>G* (rs1137101) genotyping using PCR-RFLP.**

Lanes 3, 4,8 and 9: heterozygous; lanes 2 and 5: homozygous for wild type allele (421 bp); lanes 6 and 10: homozygous for mutant type allele (294 bp and 27 bp). Lane M contains the 100 bp DNA molecular weight marker; lanes 1 and 7 negative control.


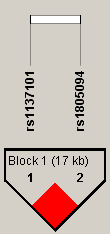


**Figure S2.**
